# Zero knowledge and high interest in the use of long-acting injectable pre-exposure prophylaxis (PrEP) among adolescent men who have sex with men and transgender women in two capital cities in Brazil

**DOI:** 10.1186/s12889-022-14134-4

**Published:** 2022-09-12

**Authors:** Leo Pedrana, Laio Magno, Eliana Miura Zucchi, Luís Augusto Vasconcelos da Silva, Dulce Ferraz, Alexandre Grangeiro, Marcelo Castellanos, Sandra Assis Brasil, Inês Dourado

**Affiliations:** 1 Instituto de Saúde Coletiva, Universidade Federal da Bahia, R. Basílio da Gama, s/n - Canela, Salvador, BA CEP: 40110-040 Brazil; 2 Departmento de Ciências da Vida, Universidade Estadual da Bahia, Salvador, Brazil; 3 Programa de Pós-Graduação em Saúde Coletiva, Mestrado Profissional em Psicologia, Desenvolvimento e Políticas Públicas, Universidade Católica de Santos, Santos, Brazil; 4 Instituto de Humanidades, Artes e Ciências, Universidade Federal da Bahia, Salvador, Brazil; 5 FIOCRUZ Escola do Governo, Fundação Oswaldo Cruz, Brasília, Distrito Federal Brazil; 6 Faculdade de Medicina da Universidade de São Paulo, São Paulo, Brazil

**Keywords:** Pre-exposure prophylaxis, Long-acting injectable, Acceptability, Adolescents, HIV/AIDS

## Abstract

**Background:**

Adolescent men who have sex with men (aMSM) and transgender women (aTGW) are affected disproportionately by human immunodeficiency virus (HIV) infection. Although new methods of pre-exposure prophylaxis (PrEP), such as long-acting injectable (LAI-PrEP), have been approved by the Food and Drug Administration, their acceptability among aMSM/aTGW is not well known.

**Methods:**

Forty-eight semi-structured interviews were conducted to assess the knowledge and interest in LAI-PrEP among aMSM/aTGW enrolled in a daily oral PrEP cohort from two capital cities of Brazil since 2019.

**Results:**

Previous knowledge of LAI-PrEP remains scarce, but the high interest regarding its use has been reported. Interest in the use of LAI-PrEP is associated with eliminating the burden of daily responsibility or the risk of missing the necessary medications, lowering the costs of this method, increasing confidentiality, and decreasing the frequency of visiting PrEP clinics. The reported barriers to uptake included fear of injection, doubts on its effectiveness, side effects, and greater dependence on a health provider.

**Conclusions:**

There is an urgent need to strengthen the preventive strategies against HIV infection among the youth, enhance their knowledge and those of healthcare providers, and offer safe and new options.

**Supplementary Information:**

The online version contains supplementary material available at 10.1186/s12889-022-14134-4.

## Introduction

Long-acting injectable pre-exposure prophylaxis (LAI-PrEP) is an effective prevention method against human immunodeficiency virus (HIV) infection that involves intramuscular injection of the antiretroviral drug cabotegravir into the gluteus muscle. In clinical trials, LAI-PrEP showed higher efficacy in preventing HIV infection among adult men who have sex with men (MSM) and transgender women (TGW), with a 66% reduction in the incidence rate compared with daily PrEP with tenofovir disoproxil fumarate–emtricitabine [1].

The efficacy and effectiveness of oral PrEP is dependent on patient’s adherence to the treatment [2, 3]; a few existing studies indicated that adherence to daily oral PrEP treatment remains a challenge among adolescents MSM (aMSM) and TGW (aTGW) [4]. Furthermore, studies on PrEP access, use, and effectiveness among adolescents emphasized several important challenges, including free-of-charge access for those aged below 18 years or those who do not have parental consent; barriers due to stigma; and discrimination based on sexual orientation and gender identity. The primary challenges in increasing PrEP awareness include low-risk perception and lack of knowledge on the risk factors of HIV infection and its preventive strategies among adolescents and healthcare providers [4–8].

Given these barriers and challenges, LAI-PrEP may be a promising modality for increasing the effectiveness of PrEP among adolescents. Studies conducted in the United States and South Africa indicate a high (> 80%) proportion of young adult MSM who express an interest in using injectable PrEP [9, 10]. Interest in the use of LAI-PrEP is associated with a higher educational level [9–11] and a higher number of sexual partners [10]. Among the primary aspects that motivate such interest are ease of use and longer duration of protection compared with daily or on-demand oral PrEP [11]. On the contrary, concerns about the degree of protection and adverse effects of this regimen in the short and medium term were also indicated [10].

The epidemic of HIV infection in Brazil disproportionately affects the MSM and TGW [12]. Recent systematic reviews of studies conducted in South America [13], specifically in Brazil [14], have reported an increase in the incidence of HIV infection among young MSM. Two studies with large samples of MSM residing in urban centers in Brazil in 2009 and 2016 showed a significant increase in the overall prevalence of HIV infection, particularly among those aged > 25 years [15]. Another study also reported behavioral changes that increase the risk of exposure to HIV among young individuals, such as unprotected anal sex, sex with multiple partners, and the use of illicit drugs [16]. Stigma and discrimination remain important predictors of HIV infection in TGW [17]. Despite the epidemiological scenario of HIV infection that indicates a higher burden of new HIV infections among adolescents and young men [18, 19], the use of PrEP among adolescent MSM and TGW remains low in Brazil and elsewhere.

LAI-PrEP knowledge, interest, and service implementation for aMSM and aTGW remain scarce in low- and middle-income countries. Hence, evaluating and understanding the interest of future LAI-PrEP will be key in the implementation of this new technology. In this study, we aimed to analyze the knowledge and interest in using LAI-PrEP among aMSM and aTGW.

## Methods

### Study design and population

A qualitative exploratory study was carried out as an integration of the first demonstration cohort study of the effectiveness of daily oral PrEP among aMSM and aTGW in three Brazilian capital cities (the PrEP1519 study) [20]. This analysis is based on data from the Salvador and São Paulo study sites. Individuals aged 15 to 19 years, identified themselves as MSM or TGW, were at increased risk of HIV infection, and reported living in the cities or metropolitan region of the study area were eligible for the PrEP1519 cohort study. Participants who met the clinical eligibility criteria and demonstrated interest in its use were included in the “PrEP group,” while those who used a combination of other prevention strategies, such as condom use, testing, and treatment for sexually transmitted infections, were included in the “Non-PrEP group.”

The HIV from the study sites of Salvador and São Paulo, with at least 1 month of enrolment in the PrEP1519 cohort. The participants’ profile differed based on the recruitment strategy used (community-based survey, hook-up apps, and health services): age (15–17 and 18–19 years), self-reported skin color (black, brown, indigenous, white, and yellow), “PrEP” or “Non-PrEP,” and level of PrEP adherence (PrEP adherence or PrEP non-adherence), which was determined by self-reported use of at least four PrEP pills every 7 days.

### Data collection and instruments

Semi-structured interviews were conducted between June 2019 and September 2020 by a team of interviewers previously trained in qualitative research. Two interview scripts were developed for young people in the PrEP and non-PrEP groups by collaborating with project team experts. Both scripts assessed the issues related to PrEP clinic access, sexual and HIV prevention practices, interest in using PrEP modalities (such as event driven, injectable, and subcutaneous), quality of life, and future perspectives (in general). For those using PrEP, the medication adherence, interactions with healthcare providers, and repercussions of PrEP use in their daily lives were investigated. The specific issues investigated in the non-PrEP group included reasons for refusing PrEP treatment and the performance of HIV self-testing (ANEX I – Interview Script).

To present the scope of the qualitative study, the research team received support from peer navigators – young people responsible for the participants’ care and adherence – and health providers during their initial contact with the participants, which facilitated and accelerated their access to this patient group. The participants were previously contacted via telephone or messaging apps to arrange and detail the type of interview (face to face or virtual).

In Salvador, face-to-face interviews were carried out in private rooms at the PrEP1519 clinic on the day of their routine return visit, held every 3 months. In São Paulo, the interviews were mostly conducted in a private room at São Paulo University; in other venues chosen by the participants, such as a partner non-governmental organization; or at the participant’s home. In the first half of 2020, due to the coronavirus disease 2019 (COVID-19) pandemic, some interviews (*n* = 9) were conducted virtually via telephone or messaging apps.

During the interviewers, LAI-PrEP was presented to all participants as follows: “*The injectable long-acting PrEP is an alternative to the use of a daily oral PrEP pill, and it is currently under study. The method of application is as follows: You take an injection and repeat the process every two months.”* Each participant responded to the questions related to their interest in using LAI-PrEP and the motivations behind their choice, focusing on the perceptions of the method’s advantages and disadvantages. The questions were answered based on participants’ previous knowledge (*N* = 48); however, two interviews were interrupted owing to the participants lack of interest in continuing the activity (*N* = 46).

### Data analysis

Eight team members recorded, transcribed, registered, and coded the collected data using the NVIVO12^R^ software. To reduce bias in the analysis, two strategies were used: the coding process for each interview was double checked by two different researchers, and two weekly meetings between and within both teams of researchers were held to discuss and validate the process and the results based on the individual category.

The prospective interest in using LAI-PrEP was categorized as either “Yes, I would use it” or “No, I would not use it” when clearly expressed; additionally, the subcategories “yes, maybe” and “no, maybe not” were established to contemplate the nuances of less favorable and unfavorable answers. The level of interest in using LAI-PrEP was compared with that in using daily oral PrEP, even in participants who were PrEP naive. In addition to the interest in using LAI-PrEP, the responses were categorized based on thematic content analysis [21], considering the advantages and disadvantages of its use, barriers, and facilitators predicted and prospected regarding the continuity and adherence to LAI-PrEP. The emic categories identified from the interviews were integrated in the coding categories based on the objectives of the study.

The analysis was performed in accordance with the ethics of care to preserve the identity and psychophysical integrity of the participants. The codes used to ensure participant anonymity enabled the identification of gender identity (MSM or TGW), age, usage experience, level of adherence (PrEP adherence or PrEP non-adherence), or non-use of PrEP (Non-PrEP), and the city where the interview was conducted (São Paulo or Salvador).

The PrEP1519 study was approved by the Research Ethics Committee of the World Health Organization (Protocol ID: Fiotec-PrEP Adolescent study), the School of Medicine of the University of São Paulo (#3.082.360), and the Collective Health Institute of the Federal University of Bahia (#3.224.840) and complied with the Brazilian National Health Council ethics resolutions 466/2012 and 510/2016. Written informed consent was obtained from adolescents aged ≥18 years. Participants aged below 18 years and their legal guardian signed an informed consent form. Meanwhile, participants from the São Paulo site provided court authorization waiving the need for parental consent as a form of agreement to their participation in the study.

## Results

### Participants’ profile

Approximately half of the participants (*N* = 48) were from the cities of Salvador (*n* = 25) and São Paulo (*n* = 23), with higher proportions of aMSM (*n* = 39) than aTGW (*n* = 9). The self-reported skin color of majority of respondents was black or brown (*n* = 35). Most of the respondents were aged 18–19 years (*n* = 41), self-reported as gay (*n* = 30), had completed or were attending secondary school (*n* = 33), and used daily oral PrEP (*n* = 36) with high level of adherence (*n* = 20) at the time of the interview (Table 1).

**Table 1:** Description of the participants and previous knowledge of and interest in LAI-PrEP

Characteristics	N	%
**Population group**		
MSM	39	81.3
TGW	9	18.8
**Sexual orientation**		
Homosexual	30	62.5
Bisexual	10	20.8
Heterosexual	7	14.6
Pansexual	1	2.1
**Race or skin color**		
Black	25	52.1
Brown	10	20.8
Indigenous	1	2.1
White	12	25.0
**Age**		
16	1	2.1
17	6	12.5
18	10	20.8
19	21	43.8
20	10	20.8
**Schooling**		
High school (complete)	7	14.6
High school (attending)	12	25.0
High school (incomplete)	1	2.1
Middle school (complete)	14	29.2
Middle school (attending)	10	20.8
Middle school (incomplete)	1	2.1
Elementary school (complete)	3	6.3
**PrEP use and adherence level**		
High PrEP adherence	20	41.7
Low PrEP adherence	16	33.3
Non-PrEP user	11	22.9
Missing	1	2.1
**Prior knowledge of LAI-PrEP**		
Yes	3	6.3
No	45	93.8
**Interest in using LAI-PrEP**		
Yes, I would use it	32	66.7
No, I would not use it	10	20.8
Yes, maybe	1	2.1
No, maybe not	3	6.3

The participants reported a low socioeconomic status and had living conditions that rendered it impossible to achieve their desires, dreams, and goals in the educational- and job-related aspects:

*“My family's financial situation is not good... my mother is currently unemployed, it’s my father who works. (...) It ’s not that life where you can go buy the things you feel like, but we manage to support ourselves. (...). I think this has influenced me a lot to try to study and have something in the future.”* (#56, White, MSM; # 17, PrEP: non-adherence, SSA)

### Knowledge and interest in using LAI-PrEP

Few respondents stated having some previous knowledge about or had heard of LAI-PrEP (Table 2), which was imparted to them by friends/peers who had been using it as participants in an LAI-PrEP demonstration study in São Paulo or by healthcare providers from PrEP services in São Paulo. One participant claimed that they had obtained information on social media through a digital influencer. However, the information was not in agreement with the available scientific knowledge, as these participants had doubts about LAI-PrEP effectiveness, similar to those who were informed by the interviewer. Most participants reported an interest in using LAI-PrEP. In fact, the advantages of using this method were mentioned more frequently than the disadvantages (Table 2).

**Table 2:** Interest in using LAI-PrEP by study variables

Variables	Yes		Maybe, yes		No		Maybe not		Total
MSM	30	77%	1	3%	5	13%	3	8%	**39**
TGW	2	22%	0	0%	7	78%	0	0%	**9**
Advantages	51	81%	3	5%	8	13%	1	2%	**63**
Disadvantages	17	45%	3	8%	17	45%	1	3%	**38**
Experience in PrEP use	25	71%	1	3%	7	20%	2	6%	**35**
No experience in PrEP use	7	64%	0	0%	3	27%	1	9%	**11**
PrEP Adherence	11	55%	0	0%	8	40%	1	5%	**20**
PrEP Non-adherence	14	93%	1	7%	0	0%	0	0%	**15**

### Perception of the advantages and facilitators of using LAI-PrEP

The perceived advantages of LAI-PrEP were as follows: the lower frequency of intake, lower chance of discontinuity, and practicality of its use. Furthermore, some participants reported the greater possibility of ensuring confidentiality among family members regarding the use of PrEP and maintaining trust in the healthcare providers in administering this treatment, considering the risks and consequences of inappropriate self-administration of injections as in the case of pregnancy prevention.

*“I think there should be a method that (...) the girl takes an injection every month to avoid the risk of having a child. It would be nice to have, like a month-long PrEP injection, instead of having to pill every day.”* (#17, White, MSM, # 17, PrEP-adherence, SSA)

#### Benefits associated with lower frequency of drug use: getting rid of a daily obligation

The frequency of LAI-PrEP use was relatively low compared with that of oral PrEP, especially when the latter was considered as a “commitment,” an “obligation,” or a “discipline,” which is considered as unwanted and inappropriate for the routine of an adolescent who were perceived as “rebels” and disliked following strict rules, especially during vacation and travel periods.

*“Man, injecting (that) would make me more relaxed. Because taking medicine every day … besides forgetting, I hate, it is like punishment, an obligation. I rebel against my obligations. I hear this a lot, and I am rebellious. I do not like obligations.”* (#46, black, MSM 19, non-PrEP, SSA).

*“There are people who give up on PrEP because it has to be used every day, at the same time, and it requires a certain type of discipline, right? For you to keep taking it, and many people are not willing to do that (...) although I have this discipline, to be able to take it every day like that, (with LAI-PrEP) it would be less of a concern, for example, one less task a day, not having to take PrEP.” (#49, Black, MSM, # 19, PrEP non-adherence, SSA)*

*“For example, on long trips, you sometimes kind of disconnect from obligations (...), from chores, a PrEP (...), that you do not need to take every day and then I will go back again to having it injected, that would be interesting.”* (#49, Black, MSM, # 19, PrEP non-adherence SSA)

#### Solution against forgetting to take the medication

Another perceived advantage of LAI-PrEP over daily oral PrEP is the lower chance of missing a dose of the medication. Eliminating the daily commitment to take PrEP pills was perceived as an opportunity to get rid of the worries and/or fears of missing a medication and, consequently, may reduce the possibility of PrEP discontinuation or PrEP overdose (i.e., taking more than one pill a day as a consequence of forgetting to take the previous pill).

*“I would use it because it would be good (avoiding) having to use the medicine every day, (so) this form of injection would be great. Not to have an excuse like: “Oh, I forgot to take the medicine,” I did not take it, that's why it happened without a condom, and I got HIV. Got it?”* (#43, Black, MSM, # 19, PrEP non-adherence, SSA)

One of the participants pointed out the possibility of unpredictable and/or everyday situations in which adolescents could be involved in, such as participating in long parties or even organizing social events such as going to the beach on Sundays, which could interrupt the daily regimen.

*“If I go out somewhere, for example, if I go to the beach on Sunday, and I have to take the medicine at night and I am not back in time, or if I go out and go somewhere else, understand? Or the party goes on overnight and I forget to take it and such.”* (#46, black, MSM; # 19, non-PrEP user, SSA)

Among participants who were not adherent to the PrEP regimen and were only interested in using the LAI-PrEP, forgetfulness and difficulty in incorporating this new habit into their usual routine were among the main reasons for discontinuing PrEP.

*There was a time when I was not working out; I resumed working out, and every day I was taking it when I arrived at the gym. I was in doubt about whether I had taken it. Because I took it and I was in doubt afterwards, did I take it? Did I or did I not?”.* (#45, White, MSM, # 20, PrEP non-adherence, SSA)

*“Sometimes I go out and forget it at home. I go out with my backpack a lot, sleep over at my aunt's house, take my clothes, take my backpack, and sometimes forget to take my PrEP. I once took an empty vial because I had two small vials, for two months, and I took the empty vial with me and left the full one at home’* (#45, Brown, MSM, 20, PrEP-Non-adherence, SSA)

#### Perceived practicality of LAI-PrEP: greater independence from the health center and lower costs

For some participants, lower intake frequency was also associated with the idea of having greater independence from the PrEP clinic, especially considering the high transportation costs for adolescents and the required commuting time, particularly for adolescents who lived far from the clinic.

*“I thought it was cool and since I live far away it is even better. Like, I go there, do the exam and such, and then I take it, then after months I go back there again, it’s even better, you know? (...). And like, it's not every time I have the money, right?”* (#21, Brown, MSM, # 17, PrEP adherence, SP)

Some participants with low adherence to PrEP reported that the displacement to pick up the medication monthly was considered a barrier, possibly related to their socioeconomic vulnerability and the change in residence, which were shown in the interviews’ undertones.

*“I stopped using it because I stopped living downtown [the same location of the PrEP clinic], so it was a bit difficult, you know? Like, finding the time to go to the place where I used to pick up [PrEP].”* (#49, Black, MSM, # 19, PrEP non-adherence, SSA)

Other participants with low adherence to PrEP reported having difficulty swallowing PrEP pills and found LAI-PrEP to be a better option despite having concerns about receiving an injection.

*“Needles, those things... I don't know... I don't know... So, maybe, maybe, I would use it... It's bad, even, to swallow that pill. I have a hard time swallowing that thing, you know?”* (#49, Black, MSM, # 19, PrEP non-adherence, SSA*)*

#### LAI-PrEP–others–target populations: “crazy friends,” “sex workers,” and “always forgetful”

Some of the participants not interested in using LAI-PrEP reported advantages for people they knew from their circle of friends, stressing the importance of feeling free from the commitment to use PrEP daily. Some target populations were more suitable for LAI-PrEP than that for oral PrEP (e.g., those who had a hectic and/or busy life and those who had sex with several partners, including sex workers):

*“[LAI-PrEP] It is more suited for people who forget a lot, are very busy, work in the world, sex work too, because they sleep a lot outside the home and forget to take their medicine [...]. I have a friend who is a sex worker. [...]. She is very forgetful and understands?”.* (#83, white, TGW, 18, PrEP adherence, SSA)

*“Oh yes, I would advise it. There are some friends of mine, for Heaven’s sake, for God's sake, they are up to no good. My trans friends, the ones who are married.”.* (#89, indigenous, TGW, 19, PrEP-non-adherence, SP)

*“Oh, I don't know anyone, but for people who do porn, in the porn industry, people who have relationships with several partners, sex workers...”.* (#62, White, MSM 19, Non-PrEP users, SSA)

### Perception of disadvantages and barriers to LAI-PrEP use

Lack of confidence in the efficacy and fear of pain caused by the injection are some of the disadvantages. Furthermore, some respondents reported greater dependence on the PrEP clinic due to the need for a periodic injection administered by a healthcare provider, unlike the use of oral PrEP. Additionally, some aTGW raised concerns about the interaction between LAI-PrEP and feminizing hormone therapy.

#### Fear of injection, doubts about handling and storage of LAI-PrEP

Fear of injection was regarded as a possible barrier to LAI-PrEP use, based on the testimonies of those who were less interested in this modality. Some participants were also concerned about the consequences of incorrectly self-administering the injections.

*“I would think it's bad because there are a lot of people who don't like to take injections. I would rather take it. I prefer to take a pill every day.”.* (#17, White, MSM, # 17, PrEP adherence, SSA)

*“The disadvantage I think would be the handling, someone handled it wrongly, someone applied it wrongly, jabbed wrong, you know? Sometimes, I think about the contraceptive that sometimes women take at home or at the pharmacy, and it can happen that they are wrongly applied and I think that it can also happen in this model.”* (#62, White, MSM 19, Non-PrEP users, SSA)

A young MSM also expressed concerns on the storage conditions of the medication used in LAI-PrEP, regarding it as more “complicated.”

*“And the means also, like, for preserving it, it's not, like, all-encompassing. As I do not know, an injection is a liquid, it will have to reach a certain temperature, and it will have to be kept in a given environment. Therefore, this process is complicated. So, I think it's complicated.”.* (#16, Black, MSM; # 17, PrEP adherence, SP)

#### Little confidence in the efficacy, doubts about dosage, and fear of side effects

Doubts on the efficacy and safety of LAI-PrEP were another disadvantage, even among those who had some previous knowledge and who expressed little or no interest in this regimen: *“My doubts are more scientific. Is it efficient?”. (#25, black, MSM, 20, PrEP-adherence, SSA); “I knew injectable [PrEP] existed. But I did not know if it was 100% safe.” (#22, Brown, MSM; 20, non-PrEP users, SP).*

However, the comparison of dosage concentrations between the two regimens is a central point underlying the unfavorable positions due to the unpredictable and unknown side effects.

*“Maybe, I don't know, the side effects, all of a sudden. You sum up the 60 days in one go. Sixty pills at once.”* (#46, black, MSM; # 19, non-PrEP user, SSA)

*“It's very good, but it's scary because it's a huge dose of medicine you are taking, right? As we do not, we are not at the mercy of knowing if you are going to have an allergic reaction. It's like the injectable contraceptive, like it's a very large dose that you are taking, so it varies from body to body. [...]. Therefore, there is the insecurity of not knowing if this high drug dose will cause something to your body, because when you take it assiduously, at least it will be something in the long term. If you take a very high dose, once, every two months, you are at the mercy of some kind of side effect, it comes faster.”.* (#22, Brown, MSM, # 20, non-PrEP user, SP)

#### Fear of forgetting and worry of discontinuation

For some, the risk of forgetting to go to the clinic to receive the injection appeared to be a disadvantage of LAI-PrEP. This greater autonomy and even the practicality of “taking daily oral PrEP anywhere” seemed to be linked to the perception of greater safety associated with this modality:

*“Ah, I think the daily PrEP, because, for example, you're going to go there* [health service]*, sometimes you forget too, sometimes you forget... Then you forget, now not anymore, PrEP daily, the small vial of medicine is already in your house, for you to take.”* (#21, brown, MSM; 17, PrEP adherence, SP).

*“Because it could be that I had the injection today, but I did not schedule anything, but I do not know, a month and a few days, I do not know, a month and twenty days from today I have to travel somewhere and I will be gone for 30 days. So, I was past the deadline for taking the medicine. Then, I did not take my medicine and it happens that … for example … I was on this trip and it happened that I ended up having sex with someone there. So, I am not going to be “protected” if I am using a condom, yes, I am. However, if you do not have it at the moment, how does it happen? So, I think it is cool, but not cool. Because the medicine's facility is that you can take it everywhere, right.”* (#27, black; MSM, 17; PrEP adherence, SP)

#### Dependence on the PrEP clinic and health professional

Others also explained the excessive dependence on the PrEP clinic or on the healthcare provider to use injectable PrEP and, therefore, had lower autonomy on the routine use of PrEP:

*“So, if you see that thinking broadly now, if you see that every time it requires you to go there every two months, it requires a certain periodicity, for example, for you to do that, I think it would be a little complicated.”* (#21, brown; MSM, 17; PrEP adherence, SP)

#### Safety and comfort of taking daily oral PrEP

In the group with lower interest in using LAI-PrEP, we also found the third factor linked to the adaptation to daily oral PrEP, which some considered as the *“Comfort zone.”* The use of injections on a regular basis, every 2 months, was an aspect that need to be considered in this PrEP modality.

*“It's that comfort zone issue, right? I will not complain about the pill. Injection is something like... the needle, this thing... I do not know... I do not know... I am still fixed on this issue with the needle, right?”.* (#39, black, MSM; # 18, PrEP-nonadherence, SSA)

*“Well, as I like to take medicine... it's not that I like it, but I have the habit of taking it, so it does not make any difference to me, taking an injection or a pill.”* (#83, white, TGW, 18, PrEP adherence, SSA)

#### Interference with feminizing hormone therapy

The doubt and suspicion about the incompatibility of LAI-PrEP with feminizing hormone therapy were also mentioned, which might decrease the interest in LAI-PrEP use among TGW:

*“And I also know that there are other types of PrEP, such as injectable PrEP. However, even when talking to the infectologist, she said there is a risk of the injectable part, which is not recommended for trans women, precisely because it conflicts with hormone therapy.”* (#37, white, TGW, 19, PrEP adherence, SP)

## Discussion

Despite the limited knowledge on injectable PrEP, the participants’ perceptions were supported by their experience with the use of daily oral PrEP through their participation in the PrEP1519 cohort. There is an ongoing prevention-learning process in which the main motivations that guide the interest of adolescents to learn about and to use injectable PrEP allow the development of a more simplified technique to use this method and provide freedom from the “strings” attached to taking daily oral PrEP. In addition, perceptions that reduce the interest of adolescents in this method are mainly linked to doubts about its effectiveness and safety, as well as the acknowledgement that the daily oral regimen meets their prevention needs.

Our findings show some specificities and congruence in relation to the findings of qualitative and quantitative studies that analyzed the use of alternative methods for the daily usage of PrEP pill. In this sense, the profile and identification of participants as vulnerable adolescents is the highlight of the study; looking at the panorama of quali-quantitative research, only a few studies contemplate this target audience [22–26], consider transgender [27, 28], consider previous PrEP usage experience [29], determine the level of PrEP adherence [26], or take into account the first experiences of using LAI-PrEP [23, 30].

The interest in LAI-PrEP shown by most of the participants in our study mitigates the widespread idea in the literature of a trend toward greater interest in and accessibility of this method among adult men with high education levels. In fact, only Biello et al. [26] studied more vulnerable categories of young people (e.g., youth with a higher frequency of casual relationships), including sex workers and those who use hook-up apps. They also emphasized the need to expand the focus on the adolescent and young key population (AYKP) to improve the effectiveness of LAI-PrEP.

In our study, participants who had experienced using daily oral PrEP seemed to be more interested in receiving LAI-PrEP compared with those who did not have such experience, as also shown by Biello et al. [26] in groups with greater vulnerability (by race/skin color, socioeconomic status, and level of education).

With regard to the motivations for using LAI-PrEP, the lower intake frequency opens up a range of possibilities linked to the reduction of responsibility and commitment to observing the daily PrEP routine that barely fits into the lifestyles and dynamics of sexual encounters of adolescents and youth. These aspects also motivated those who are concerned about skipping doses [23, 30, 31] and reducing adherence [22], mainly due to the difficulty in remembering or impediments and unforeseen events. Moreover, LAI-PrEP can be used as a strategy to manage HIV-, sexual-, and gender-related stigmas. Among long-acting medications, PrEP is regarded as a method that provides greater discretion and confidentiality, especially for those living with family members resistant and/or intolerant to PrEP, homosexuality, transsexuality, and cross-dressing people, which has been pointed out in several studies [23–26, 30, 31], although it has been less frequently mentioned in our study.

The perception of the potential simplification of access and the use of PrEP linked to the lower frequency of LAI-PrEP intake is a contradictory issue. For some participants, the use of LAI-PrEP would imply a lower frequency of visiting PrEP clinics, as indicated by some studies [23, 29, 32], including a reduction in costs [30]. For some participants, this method imposes a complete dependence on the health service to perform the PrEP injection, thus configuring itself as an interest-reducing factor, as pointed out in several studies [24, 26, 30, 32]**.**

Among the fears that reduce interest in using LAI-PrEP are related to its administration–that is, fear of the invasive and painful modality of injection [22, 24, 27, 29, 31]. In this regard, as evidenced by Patel et al. [22], the lack of information implies that the LAI-PrEP is compared or associated with other injectable drugs with a preventive ability, such as vaccines or contraceptives. In addition, the lack of information on the characteristics and effects of this drug reduced the confidence in LAI-PrEP’s protection and efficacy [24, 29] and increased the individual’s fear on the effects of using a higher medication dosage compared with oral PrEP [22, 24, 26, 27, 30]. Among the participating TGW, this concern or skepticism about the effects of LAI-PrEP stems from the possible interference with feminizing hormone therapy ( [27, 28].

As the participants were less knowledgeable about LAI-PrEP, including those who already knew or used daily oral PrEP, building an informed understanding of the use of LAI-PrEP involves promoting and investing in information about this method as a strategy for generating demand and care. This can also be related to the trust and comfort with the habit of taking medications daily, which becomes a disadvantage for LAI-PrEP, as demonstrated in other studies conducted among young MSM in North America [22, 25].

In fact, our study found that for some adolescents, the frequency of use is linked to the perception of continuity and effectiveness of daily oral PrEP’s power (i.e., safety and protection); therefore, the greater intake interval characteristic of LAI-PrEP is considered a problematic issue.

### Study limitations

The limitations outlined below must be considered when interpreting the results and discussion.

The age of the participants did not fully represent the age groups conventionally considered as adolescents (12–18 years) and young people (15–24 years) in Brazil. Hence, future studies should analyze younger age groups, who have limited access to healthcare and HIV prevention services.

Owing to the ongoing COVID-19 pandemic, the use of a combination of face-to-face and virtual interviews can affect the data quality and uniformity.

The expression of interest in LAI-PrEP usage was considered in this study to enable the comparison between the experience of using daily oral PrEP and the prospective interest in using LAI-PrEP. Other studies will be able to devote more attention to the different positions, subtle differences, and nuances between desires, interests, intentions, and motivations for use depending on the daily reality of young people. Moreover, at the conduct of this study, the injectable PrEP drug was still in the trial phase. In fact, information about LAI-PrEP is limited and essentially focused on the modality of use and scientific literature.

## Conclusions

Our study highlights the importance and urgency of implementing demand creation to promote knowledge and information about PrEP modalities that can reach AYKP, as well as expand access and availability to adolescents and youth who are already familiar with the use of oral PrEP, with a focus on the greater vulnerability and greater difficulty in adapting its daily use. Thus, the PrEP portfolio can be improved and perceived as a preventive and effective care technology for all young people and adolescents at increased risk of HIV infection, considering their specificities and practices. However, the use of PrEP among adolescents and youth should be take place under the guidance, support, and monitoring of healthcare professionals who can provide a set of attention and care to the sexual health of the young person in order to optimize the effectiveness of this method, considering that the use and demand for PrEP and its modalities are not fixed or stable, and may change in different contexts or moments in life.

## Supplementary Information

**Additional file 1: Annex I.** Interview Script.

## Data Availability

The dataset analysed in this study are not publicly available because are used under license for the current study, but are available from the corresponding author on reasonable request.
