# Supplementary material for: Zero knowledge and high interest in the use of long-acting injectable pre-exposure prophylaxis (PrEP) among adolescent men who have sex with men and transgender women in two capital cities in Brazil
Source: BMC Public Health. 2022 Sep 12;22:1728. doi: 10.1186/s12889-022-14134-4 (PMC9465661; doi:10.1186/s12889-022-14134-4)
Supplement: Supplementary file 1 — Additional file 1: Annex I. Interview Script. [file 12889_2022_14134_MOESM1_ESM.docx]

# ANNEX I

# Interview Script

# IDENTIFICATION

Date of interview ____/_____/______

Name ________________________________________

Medical record number (PID): ___________.

Time in the project ( ) Recruitment ( ) Attachment ( ) Retention

Age: __________

Race/skin color (self-declared):________________________________________________

Gender identity and sexual orientation: _______________________________________________

Education: __________________________________________________________

What do you do (work, profession, or study)? ____________________________________

Where do you live and with whom? ___________________________________________

Do you agree to be contacted again? Yes ( ) No ( )

WhatsApp: ____________________ Facebook: _______________________________

Do you agree in the recording of the interview? Yes ( ) No ( )

(If “No,” thank the participant and do not conduct the interview.)

How long have you used PrEP? ____________________________________________

Name of interviewer: _______________________________________________

Place: Salvador ( ) São Paulo ( )

QUESTIONS

- Tell me a little about yourself.
- Tell me how you arrived at the project.
- What were the reasons that made you participate in the project?
- Did you have difficulty entering the project? If so, can you talk about these difficulties?
- What makes you stay on the project?
- Tell me an interesting/good experience you had on the project.
- What makes it difficult for you to remain on the project?
- Tell me a bad experience you had on the project.
- How has the experience of using PrEP affected in your daily life?
- Who did you tell you that are using PrEP? What was that like?
- How is your sex life while using PrEP?
- What is it like to take the PrEP pill every day?
- What do you think are the biggest difficulties or inconveniences in using PrEP?
- Would you advise a friend to use PrEP? Why?
- Could you tell me what you think about your future life and expectations?
- Have you heard of other ways to use PrEP? What were they? Could you tell us a little more than what you have heard?
- Are you familiar with injectable PrEPs? What are the advantages and disadvantages of injectable PrEP?
- In what situations would you use injectable PrEP?

This was the end of the interviews. Is there anything you’d like to add or any topics that I have not covered that you'd like to talk about?

Thank you for your participation!
